# Response Rate and Survival at Key Timepoints With PD-1 Blockade vs Chemotherapy in PD-L1 Subgroups: Meta-Analysis of Metastatic NSCLC Trials

**DOI:** 10.1093/jncics/pkab012

**Published:** 2021-01-27

**Authors:** Johnathan Man, Jared Millican, Arthur Mulvey, Val Gebski, Rina Hui

**Affiliations:** 1 Department of Medical Oncology, Crown Princess Mary Cancer Centre, Westmead Hospital, Sydney, NSW, Australia; 2 Sydney West Translational Cancer Research Centre; 3 University of Sydney, Australia

## Abstract

**Background:**

Expression of programmed cell death ligand 1 (PD-L1) on tumor cells with or without immune cells is widely reported in clinical trials of programmed cell death receptor 1 (PD-1) blockade in metastatic non-small cell lung cancer. Various cutpoints have been studied.

**Methods:**

We performed a systematic search of MEDLINE, EMBASE, and conference proceedings up to December 2019 for randomized and nonrandomized clinical trials of anti-PD-1 or anti-PD-L1 monotherapy in metastatic non-small cell lung cancer. We retrieved data on objective response rate (ORR), 1-year and 2-year progression-free survival (PFS), and 2-year and 3-year overall survival (OS) in various PD-L1 subgroups. Results were pooled and analyzed based on different cutpoints, with nonrandomized comparisons made with pooled chemotherapy outcomes.

**Results:**

A total of 9810 patients in 27 studies were included. In treatment-naïve patients, benefits with PD-1 blockade over chemotherapy were seen in ORR in patients having PD-L1 50% or greater, in 2-year OS for PD-L1 1% or greater, and in 1-year PFS, 2-year PFS, and 3-year OS for unselected patients. First-line PD-1 blockade compared with chemotherapy demonstrated higher ORR, 2-year PFS, and 3-year OS if PD-L1 was 50% or greater; lower ORR, higher 2-year PFS, and similar 3-year OS if PD-L1 was 1%-49%; and lower ORR, similar 1-year PFS, and lower 2-year OS if PD-L1 was less than 1%. In previously treated patients, PD-1 blockade demonstrated similar or superior outcomes to chemotherapy in all PD-L1 subgroups.

**Conclusions:**

PD-L1 should guide the choice of PD-1 blockade vs chemotherapy in treatment-naïve patients. In previously treated patients, PD-1 blockade provides a favorable outcome profile to chemotherapy in all PD-L1 subgroups.

The most common cause of cancer death is lung cancer, with an estimated global incidence of 1.8 million and mortality of 1.6 million per year (1). Non-small cell lung cancer (NSCLC) without oncogenic driver mutations harbors a high burden of somatic mutations (2), which act as antigenic targets for host immunity. Evasion of immune destruction is a key mechanism of lung cancer development and was described as an emerging hallmark of cancer by Hanahan and Weinberg in 2011 (3). The relationship between tumor cells and host immunity usually favors tumor elimination or an equilibrium between immune destruction and tumor growth, but when tumor cells progress to immune escape, these cancer cells overcome host immunity, protect themselves with an immunosuppressive environment, and metastasize with fatal consequences (4).

Interaction between programmed cell death receptor 1 (PD-1) on T cells and programmed cell death ligand 1 (PD-L1) on tumor cells plays an important role in immune evasion. PD-1 and PD-L1 inhibitors, herein referred to as PD-1 blockade, have been established as standard of care in both treatment-naïve and previously treated patients with advanced NSCLC (5,6). Some patients experience durable tumor responses, but others derive no clinical benefit, highlighting the importance of identifying biomarkers to improve patient selection. PD-L1 expression on tumor cells with or without its expression on immune cells remains the most reported association with antitumor activity of PD-1 blockade.

PD-L1 expression is a continuous variable ranging from 0% to 100% based on the percentage of tumor cells in a tissue specimen that display partial or complete PD-L1 membrane staining of any intensity (7,8). In some assays, the percentage of tumor-infiltrating immune cells with PD-L1 staining is also measured (7,8). PD-L1 is often reported in a dichotomous fashion, where tumors are defined as either “PD-L1 high” or “PD-L1 low” based on a selected PD-L1 cutpoint. The uncertainty regarding which cutpoint should be chosen is demonstrated by the variation in cutpoints used in different studies, from 1% in some trials to 90% in others (9,10). Another important consideration is heterogeneity among different PD-L1 assays. Various pharmaceutical companies have each developed PD-1/PD-L1–inhibiting drugs with different corresponding PD-L1 assays. Results from the Phase I and II BluePrint Study, which compared the different assays, suggested that the Dako 22–8, Dako 22C3, and Ventana SP263 assays used in nivolumab, pembrolizumab, and durvalumab studies, respectively, had high concordance with each other (11). However, the Ventana SP142 assay used in atezolizumab studies had fainter staining on tumor cells and therefore produced lower scores, but in turn supplemented its scoring by including immune cell staining. The Dako 73–10 assay used in avelumab trials had stronger staining on tumor cells and therefore produced higher scores (11).

The efficacy of different PD-1–blocking agents has not been compared head to head. Nevertheless, pembrolizumab, nivolumab, and atezolizumab have all displayed similar superior efficacy over docetaxel in previously treated NSCLC patients (9,12–15), and meta-analysis has found no statistically significant observable difference between PD-1 inhibitors and PD-L1 inhibitors (16).

There are accumulating published data with longer follow-up on the relationship of PD-L1 expression with response rate, and progression-free survival (PFS) and overall survival (OS) at key time points in NSCLC patients. We conducted this meta-analysis in advanced NSCLC patients treated with PD-1 blockade, focusing on the impact of PD-L1 cutpoint choice on objective response rate (ORR), 1-year PFS, 2-year PFS, 2-year OS, and 3-year OS compared with the outcomes from chemotherapy. The aim of this meta-analysis was to identify the cutpoint at which PD-1 blockade provided superior outcomes to chemotherapy, and specifically compare PD-1 blockade with chemotherapy in the subgroups of PD-L1 less than 1%, 1%-49%, and 50% or greater.

## Methods

### Search Strategy and Risk of Bias

A systematic search of MEDLINE and EMBASE databases from January 1, 2010, to December 2, 2019, was performed (see the Supplementary Methods for the search strategy, available online) by the first author (J.M.) and checked by another author (J.Mi.). The search was supplemented with a hand search of abstracts and conference proceedings with the cutoff date of December 2, 2019. Studies meeting the following criteria were included: 1) phase I, II, or III human clinical trial; 2) reported results about stage IV NSCLC alone; 3) inclusion of PD-1 or PD-L1 inhibitor monotherapy arm; 4) reporting of PD-L1 subgroup analysis; 5) reporting of first- and second-line patients separately; 6) exclusion of studies limited only to patients with *EGFR* or *ALK* mutations; and 7) exclusion of studies limited to only patients with brain metastases.

Data were extracted by the first author (J.M.) and independently checked by 2 other authors (J.Mi. and A.M.), with any discrepancies resolved by consensus. In the presence of multiple publications of the same trial, the most updated data were chosen. Risk of bias was assessed by Cochrane Risk of Bias (17) for randomized trials and ROBINS-I Risk of Bias (18) for nonrandomized trials.

### Statistical Analysis

Analyses were performed using Microsoft Excel v15.32. We extracted data on the primary outcomes of ORR, 1-year PFS, 2-year PFS, 2-year OS, and 3-year OS in different PD-L1 subgroups of patients on PD-1 blockade monotherapy or chemotherapy. Pooled estimates and their 95% confidence intervals were calculated for the primary outcomes in each PD-L1 subgroup on each therapy. The pooled estimates were obtained as a weighted average of individual study estimates, where the study weights were determined by the inverse of the variance of the estimate in each study. This is a widely used method for pooling the results of studies and details are given in Neyeloff et al. (19). Because many studies were single arm, an analysis based on hazard ratios was not practical. Heterogeneity in the pooled estimates for each staining group was calculated by Cochran’s Q test. A fixed effect method for the variance of the pooled estimates was used.

Pooled estimates of primary outcomes were then compared between the different therapies using χ^2^ tests. All comparisons were 2-tailed, with statistical significance defined as *P* less than or equal to .05. These were nonrandomized comparisons. No adjustments were made for multiple comparisons.

Cohorts of treatment-naïve and previously treated patients were analyzed separately. When extracting data for a cutpoint of interest, we used only outcomes reported in studies that were exactly consistent with the cutpoint used. For example, if data on the PD-L1 1% or greater subgroup were being pooled, outcome data reported for the PD-L1 50% or greater subgroup were not included because they may overestimate the potential benefit for the 1% or greater subgroup. When values for PFS rate or OS rate were not explicitly stated in the manuscript, these were estimated using the published Kaplan-Meier graphs. When integrating data based on the SP142 assay, which combines tumor and immune cell staining, TC0/IC0 was considered PD-L1 less than 1%, TC1/2/3 or IC1/2/3 was considered PD-L1 1% or greater, TC2/3 or IC2/3 was considered PD-L1 5% or greater, and TC3/IC3 was considered PD-L1 50% or greater.

Because chemotherapy is unlikely to be affected by PD-L1 status, comparisons of outcomes between PD-1 blockade and chemotherapy were performed between the specific PD-L1 subgroup for PD-1 blockade and all chemotherapy patients irrespective of PD-L1 status in order to optimize the power of the chemotherapy outcome estimate and allow comparisons to occur where chemotherapy arms were not reported by specific PD-L1 subgroups.

Meta-analysis was performed on all included studies. Prespecified sensitivity analyses were performed using only studies that used the Dako 22C3, Dako 28–8, and Ventana SP263 assays, which have the most comparable staining (11).

## Results

### Identified Trials

**Figure 1.**
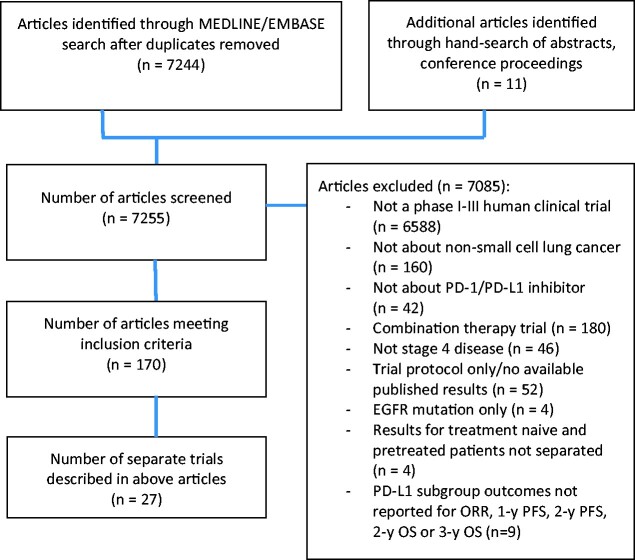
Flow diagram of included and excluded studies. EGFR = epidermal growth factor receptor; ORR = objective response rate; OS = overall survival; PD-1 = programmed cell death receptor 1; PD-L1 = programmed cell death ligand 1; PFS = progression-free survival.

A total 7255 trials were identified by the search strategy for screening (Figure 1). A total of 170 articles or conference proceedings met the inclusion criteria, many of which were updates on the same trial. In total, 27 separate trials were included (Table 1). Risk of bias assessment of included trials is provided in Supplementary Tables 1 and 2 (available online).

**Table 1.** Summary of included trials

Study (trial name)	No.^a^	Randomization	Phase	Experimental arm	Control arm	Line	Assay	Selection	PD-L1 cutpoints^b^	Primary endpoint	Minimum follow-up for survival
Carbone et al. 2017, (Checkmate 026) (20)	541	Yes	III	Nivolumab 3 mg/kg q14d	Physician’s choice chemotherapy	1st	28-8	≥1%	1, 5, 50	PFS	13.7 mo (20)
Gettinger et al. 2017, (Checkmate 012) (21, 22)	52	No	I	Nivolumab 3 mg/kg q14d	Nil	1st	28-8	All comers	1, 5, 10, 25, 50	Safety	Not specified (22)
Brahmer et al. 2015, (Checkmate 017) (12, 23–27)	272	Yes	III	Nivolumab 3 mg/kg q14d	Docetaxel 75 mg/m^2^ q21d	2nd	28-8	All comers	1, 5, 10	OS	51.6 mo (24)
Borghaei et al. 2015, (Checkmate 057) (23–27)	582	Yes	III	Nivolumab 3 mg/kg q14d	Docetaxel 75 mg/m^2^ q21d	2nd	28-8	All comers	1, 5, 10	OS	51.6 mo (24)
Rizvi et al. 2015, (Checkmate 063) (23, 28, 29)	117	No	II	Nivolumab 3 mg/kg q14d	Nil	≥2nd	28-8	All comers	1, 5, 10	ORR	56.3 mo (23)
Hida et al. 2017, (ONO-4538–05) (30, 31)	35	No	II	Nivolumab 3 mg/kg q14d	Nil	≥2nd	28-8	All comers	1, 5, 10, 50	ORR	3 y (31)
Nishio et al. 2016, (ONO-4538–06) (31, 32)	76	No	II	Nivolumab 3 mg/kg q14d	Nil	≥2nd	28-8	All comers	1, 5, 10, 50	ORR	3 y (31)
Gettinger et al. 2015, (Checkmate 003) (23, 33, 34)	129	No	I	Nivolumab 1 mg/kg, 3 mg/kg, or 10 mg/kg q14d	Nil	≥2nd	28–8	All comers	5	ORR	75.2 mo (23)
Mok et al. 2019, (KEYNOTE 042) (35, 36)	1274	Yes	III	Pembrolizumab 200 mg q21d	Physician’s choice chemotherapy	1st	22C3	≥1%	1, 20, 50	OS	Not specified (35)
Reck et al. 2016, (KEYNOTE 024) (37, 38)	305	Yes	III	Pembrolizumab 200 mg q21d	Physician’s choice chemotherapy	1st	22C3	≥50%	50	PFS	20.4 mo (38)
Garon et al. 2015, (KEYNOTE 001) (39–42)	550	No	I	Pembrolizumab 2 mg/kg q21d, or 10 mg/kg q14d or 10 mg/kg q21d	Nil	≥1st	22C3	All comers^c^	1, 50	Safety, side-effect profile, antitumor activity	51.8 mo (39)
Hersbt et al. 2015, (KEYNOTE 010) (13, 43, 44)	1033	Yes	II/III	Pembrolizumab 2 mg/kg or 10 mg/kg q21d	Docetaxel 75 mg/m^2^ q21d	≥2nd	22C3	≥1%	1, 25, 50, 75	OS and PFS	35.2 mo (44)
Theelen et al. 2019, (Pembro-RT control arm) (45)	40	Yes	II	Pembrolizumab 200 mg q21d + 8 Gy/3 Fr RT	Pembrolizumab 200 mg/kg q21d	≥2nd	22C3	All comers	1, 50	ORR	0.1 mo (45)
Nishio et al. 2019, (KEYNOTE 025) (46)	38	No	Ib	Pembrolizumab 10 mg/kg q21d	Nil	≥2nd	22C3	≥1%	1, 50	ORR, safety, tolerability	1.9 mo (46)
Levy et al. 2017, (CC-486-NSCL-001) (47)	49	Yes	II	Pembrolizumab 200 mg q21d + oral azacitadine	Pembrolizumab 200 mg/kg q21d + placebo	2nd	22C3	All comers	1, 50	PFS	Not specified (47)
Spigel et al. 2019, (IMpower110) (48)	554	Yes	III	Atezolizumab 1200 mg q21d	Platinum doublet chemotherapy	1st	SP142	≥1%	1^d^, 5^e^, 50^f^	OS	0 mo (48)
Peters et al. 2017, (BIRCH) (49, 50)	660	No	II	Atezolizumab 1200 mg q21d	Nil	≥1st	SP142	≥5%	5^e^, 50^f^	ORR	20 mo (50)
Spigel et al. 2018, (FIR) (51)	137	No	II	Atezolizumab 1200 mg q21d	Nil	≥1st	SP142	≥5%	5^e^, 50^f^	ORR	Not specified (51)
Rittmeyer et al. 2017, (OAK) (15, 52)	1038	Yes	III	Atezolizumab 1200 mg q21d	Docetaxel 75 mg/m^2^ q21d	≥2nd	SP142	All comers	1^d^, 5^e^, 50^f^	OS	21 mo (52)
Fehrenbacher et al. 2016 (POPLAR) (14, 53)	287	Yes	II	Atezolizumab 1200 mg q21d	Docetaxel 75 mg/m^2^ q21d	≥2nd	SP142	All comers	1^d^, 5^e^, 50^f^	OS	3 y (53)
Rizvi et al. 2018, (MYSTIC) (54)	746	Yes	II	Durvalumab 20 mg/kg q28d or durvalumab 20 mg/kg q28d + tremelimumab 1 mg/kg q28d for 4 doses	Platinum based chemotherapy	1st	SP263	All comers	1, 25	OS, PFS	Not specified (54)
Antonia et al. 2019, (NCT01693562) (55)	279	No	I/II	Durvalumab 10 mg/kg q14d	Nil	≥1st	SP263	All comers	25	Side-effect profile, antitumor activity	0.3 mo (55)
Garassino et al. 2018, (ATLANTIC cohorts 2 and 3) (56)	310	No	II	Durvalumab 10 mg/kg q14d	Nil	≥2nd	SP263	All comers	25, 90	ORR	3.4 mo (56)
Papadimitrakopoulou et al. 2017, (Lung-MAP SWOG S1400A) (57)	68	No	II	Durvalumab q14d	Nil	≥2nd	SP263	All comers	25	ORR	Not specified (57)
Jerusalem et al. 2017, JAVELIN Solid Tumor) (58–60)	259	No	Ib	Avelumab 10 mg/kg q14d	Nil	≥1st	73–10	All comers	1, 5, 25	Safety, tolerability	31 mo (59)
Barlesi et al. 2018, (JAVELIN Lung 200) (61)	396	Yes	III	Avelumab 10 mg/kg q14d	Docetaxel 75 mg/m^2^ q21d	≥2nd	73-10	All comers	1, 50, 80	OS	Not specified (61)
Wu et al. 2019, (SHR-1210–201) (62)	146	No	II	Camrelizumab 200 mg q14d	Nil	≥2nd	22C3	All comers	1, 25, 50	ORR	Not specified (62)

a Number of patients with published efficacy data. IC = immune cell staining; ORR = objective response rate; OS = overall survival; PD-L1 = programmed cell death ligand 1; PFS = progression-free survival; q2 wk = once every 2 weeks; q3 wk = once every 3 weeks.

b PD-L1 cutpoints with published data either in the original article or subsequent updates.

c Four cohorts enrolled PD-L1 1% or greater, 1 cohort enrolled all comers, 1 cohort enrolled PD-L1 less than 1%.

d Or IC greater than or equal to 1%.

e Or IC greater than or equal to 5%.

f Or IC greater than or equal to 10%.

Outcome data were available for 9810 patients in total, including 6950 patients on PD-1 blockade (nivolumab, n = 1107; pembrolizumab, n = 2158; atezolizumab, n = 1830; durvalumab, n = 1054; avelumab, n = 655; camrelizumab, n = 146) and 2860 patients on chemotherapy (docetaxel, n = 1525; platinum doublet, n = 1335). Eleven trials reported on treatment-naïve patients, 21 trials reported on previously treated patients, and 5 trials reported on both. All studies enrolled patients with Eastern Cooperative Oncology Group (ECOG) Performance Status of 0 or 1.

PD-L1 cutpoints used in first-line trials were 1% (7 trials), 5% (6 trials), 10% (1 trial), 20% (1 trial), 25% (4 trials), and 50% (8 trials), and cutpoints used in second- or later line trials were 1% (15 trials), 5% (12 trials), 10% (5 trials), 25% (6 trials), 50% (16 trials), 75% (1 trial), 80% (1 trial), and 90% (1 trial).

The full extracted dataset including statistical analysis can be viewed as Supplementary Material (available online for separate download).

### PD-L1 Cutpoint Required for Superiority of PD-1 Blockade to Chemotherapy

**Figure 2.**
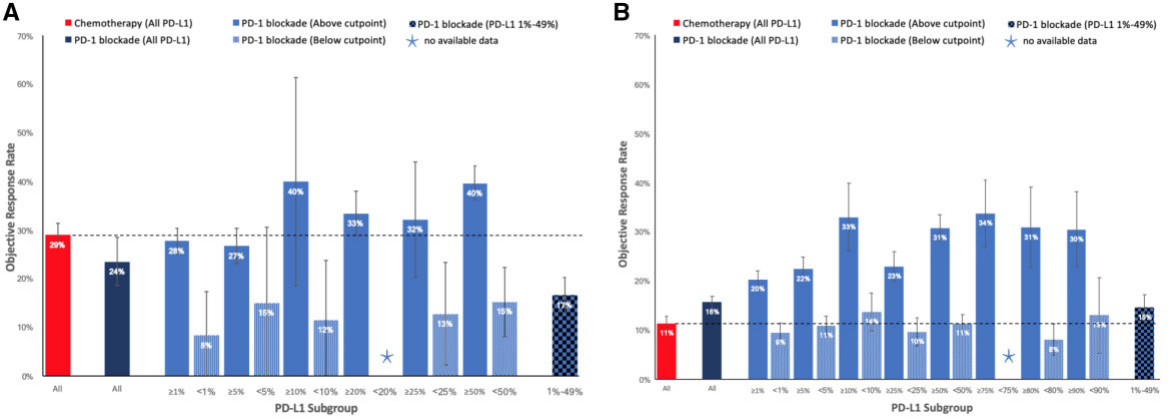
Objective response rate (ORR) of chemotherapy, or programmed cell death receptor 1 (PD-1) blockade in different programmed cell death ligand 1 (PD-L1) subgroups. **A**) ORR in treatment-naïve patients. **B**) ORR in previously treated patients. Error bars represent 95% confidence intervals for the pooled estimate.

**Figure 3.**
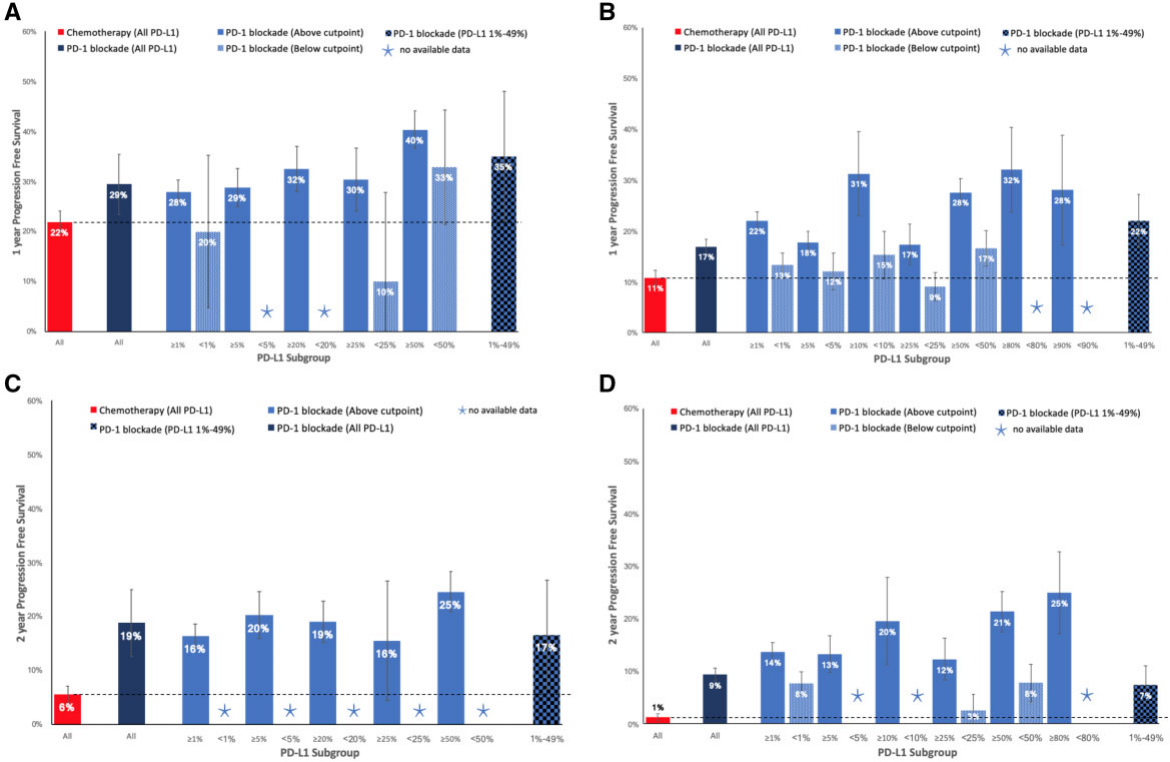
One-year and 2-year progression-free survival (PFS) rates of chemotherapy, or programmed cell death receptor 1 (PD-1) blockade in different programmed cell death ligand 1 (PD-L1) subgroups. **A**) One-year PFS in treatment-naïve patients. **B**) One-year PFS in previously treated patients. **C**) Two-year PFS in treatment-naïve patients. **D**) Two-year PFS in previously treated patients. Error bars represent 95% confidence intervals for the pooled estimate.

**Figure 4.**
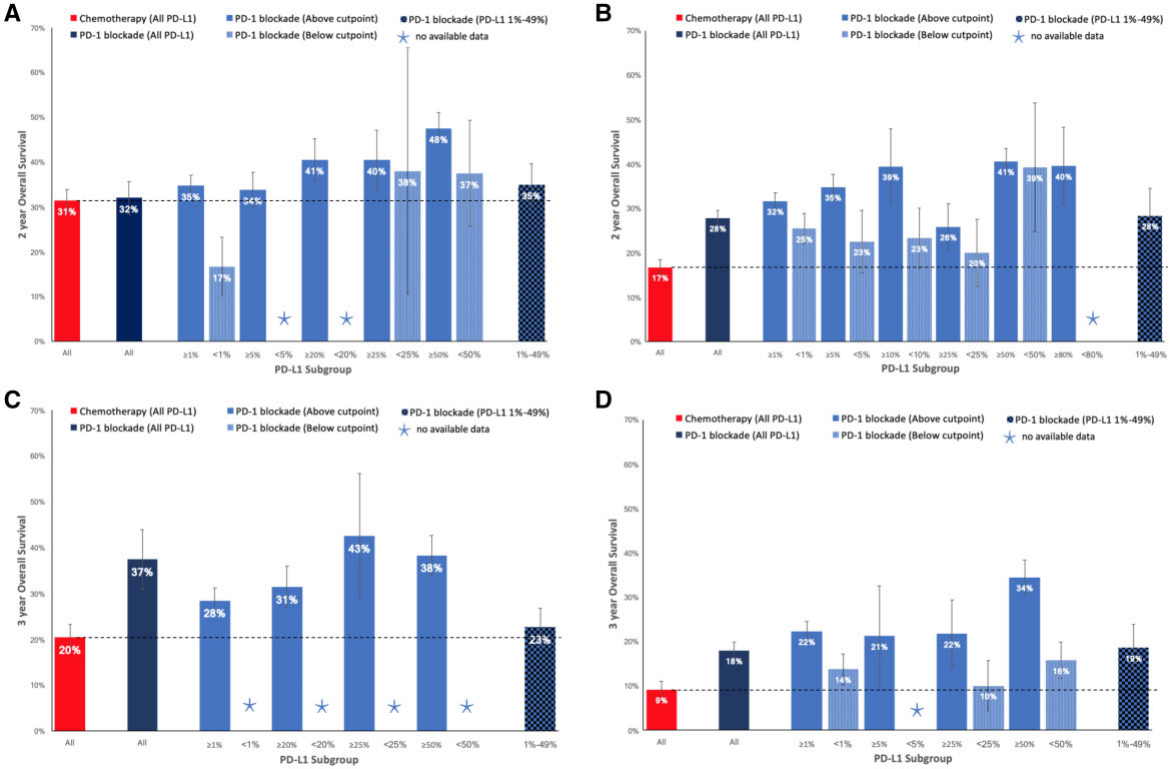
Two-year and 3-year overall survival (OS) rates of chemotherapy, or programmed cell death receptor 1 (PD-1) blockade in different programmed cell death ligand 1 (PD-L1) subgroups. **A**) Two-year OS in treatment naïve patients. **B**) Two-year OS in previously treated patients. **C**) Three-year OS in treatment-naïve patients. **D**) Three-year OS in previously treated patients. Error bars represent 95% confidence intervals for the pooled estimate. PD-1 = programmed cell death receptor 1; PD-L1 = programmed cell death ligand 1.

In the first-line setting, a 50% cutpoint was required to select patients, above which PD-1 blockade demonstrated higher ORR compared with platinum-based chemotherapy (39.7% vs 29.0%, *P* < .001) (Figure 2, A). Patients not selected by their PD-L1 status demonstrated higher 1-year PFS (29.4% vs 21.8%, *P* = .002), 2-year PFS (18.8% vs 5.6%, *P* < .001), and 3-year OS (37.4% vs 20.4%, *P* < .001) rates than chemotherapy (Figures 3, A and C, and 4C). Similar patterns were seen in populations selected above other specific PD-L1 cutpoints (Figures 3, A and C, and 4C). Treatment-naïve patients selected above the 1%, 20%, 25%, and 50% cutpoints experienced a higher 2-year OS rate with PD-1 blockade compared with chemotherapy (Figure 4, A and C).

For patients receiving second- or later line therapy, those receiving PD-1 blockade, irrespective of the level of PDL-1 expression, demonstrated better outcomes compared with docetaxel in ORR (15.7% vs 11.3%, *P* < .001), 1-year PFS (16.9% vs 10.8%, *P* < .001), 2-year PFS (9.3% vs 1.3%, *P* < .001), 2-year OS (27.8% vs 16.7%, *P* < .001), and 3-year OS (18.0% vs 9.1%, *P* < .001) rates, with larger benefits seen with increasing PDL-1 level (Figures 2, B; 3, B and D; and 4, B and D).

### Outcomes of PD-L1 Less Than 1%, 1%-49%, and 50% or Greater Subgroups

In treatment-naïve patients, outcomes for PD-L1 subgroups of less than 1%, 1%-49%, and 50% or greater were reported in 3 trials (36 patients), 3 trials (409 patients), and 8 trials (759 patients), respectively. In previously treated patients, outcomes for PD-L1 subgroups of less than 1%, 1%-49%, and 50% or greater were reported in 14 trials (10 404 patients), 10 trials (696 patients), and 15 trials (1132 patients), respectively.

### ORR in PD-L1 Less Than 1%, 1%-49%, and 50% or Greater Subgroups

As first-line therapy, PD-1 blockade resulted in objective response in 39.7% (95% confidence interval [CI] = 36.2% to 43.1%) of patients with PD-L1 50% or greater, 16.6% (95% CI = 13.0% to 20.2%) of patients with PD-L1 1%-49%, and 8.3% (95% CI = 0.0% to 17.4%) of patients with PD-L1 less than 1% (Figure 2, A). When comparing with a response rate of 29.0% (95% CI = 26.5% to 31.5%) with first-line platinum doublet chemotherapy, the ORR was statistically significantly higher in the 50% or greater subgroup (*P* < .001) and statistically significantly lower in the 1%-49% (*P* < .001) and less than 1% (*P* = .01) subgroups.

PD-1 blockade used as a second- or later line of therapy was associated with objective response in 30.7% (95% CI = 28.1% to 33.4%) of patients with PD-L1 50% or greater, 14.5% (95% CI = 11.9% to 17.1%) of patients with PD-L1 1%-49%, and 9.4% (95% CI = 7.5% to 11.3%) of patients with PD-L1 less than 1% (Figure 2, B). Compared with docetaxel in previously treated patients, which was associated with a response rate of 11.3% (95% CI = 9.9% to 12.7%), the ORR was higher with PD-1 blockade in the PD-L1 1%-49% (*P* = .03) and 50% or greater (*P* < .001) subgroups, whereas comparison between these treatment options in the PD-L1 less than 1% subgroup did not reach statistical significance.

### 1-Year and 2-Year PFS in PD-L1 Less Than 1%, 1%-49%, and 50% or Greater Subgroups

First-line PD-1 blockade was associated with 1-year PFS and 2-year PFS rates of 40.3% (95% CI = 36.6% to 44.0%) and 24.6% (95% CI = 20.8% to 28.3%), respectively, if PD-L1 was equal to 50% or greater; rates of 35.0% (95% CI = 22.0% to 48.0%) and 16.6% (95% CI = 6.5% to 26.7%) if PD-L1 was equal to 1%-49%; and 19.9% (95% CI = 4.7% to 35.2%) and no data for 2-year PFS if PD-L1 was less than 1% (Figure 3, A and C). Compared with pooled data for first-line platinum doublet chemotherapy, which results in a 1-year PFS rate of 21.8% (95% CI = 19.6% to 24.0%), and a 2-year PFS rate of 5.6% (95% CI = 4.1% to 7.1%), PD-1 blockade demonstrated higher 1-year PFS (*P* < .001) and 2-year PFS (*P* < .001) rates if PD-L1 was 50% or greater; and higher 1-year PFS (*P* = .04) and 2-year PFS (*P* = .003) rates if PD-L1 was equal to 1%-49%. If PD-L1 was less than 1%, there was little difference in the 1-year PFS rate (*P* = .80) between the 2 treatment groups.

In previously treated patients, PD-1 blockade demonstrated 1-year PFS and 2-year PFS rates of 27.5% (95% CI = 24.9% to 30.2%) and 21.3% (95% CI = 17.4% to 25.2%) if PD-L1 was 50% or greater; 21.9% (95% CI = 16.7% to 27.1%) and 7.4% (95% CI = 3.8% to 11.0%) if PD-L1 was equal to 1%-49%; and 13.2% (95% CI = 10.8% to 15.7%) and 7.7% (95% CI = 5.5% to 9.8%) if PD-L1 was less than 1% (Figure 3, B and D). Compared with second- or later-line docetaxel, which resulted in a 1-year PFS rate of 10.8% (95% CI = 9.3% to 12.2%) and 2-year PFS rate of 1.3% (95% CI = 0.6% to 1.9%), PD-1 blockade was statistically superior in each of the less than 1%, 1%-49%, and 50% or greater subgroups.

### 2-Year and 3-Year OS in PD-L1 Less Than 1%, 1%-49%, and 50% or Greater Subgroups

In patients receiving PD-1 blockade as first-line therapy, 2-year OS and 3-year OS rates were 47.5% (95% CI = 44.0% to 51.0%) and 38.3% (95% CI = 34.1% to 42.6%), respectively, if PD-L1 was 50% or greater; and 34.9% (95% CI = 30.2% to 39.7%) and 22.6% (95% CI = 18.5% to 26.8%), respectively, if PD-L1 was equal to 1%-49%. In the PD-L1 less than 1% subgroup, the 2-year OS rate was 16.7% (95% CI = 10.2% to 23.2%) and no data were available to inform the 3-year OS rate (Figure 4, A and C). Compared with patients receiving platinum doublet chemotherapy as first-line therapy, which resulted in a 2-year OS rate of 31.4% (95% CI = 29.0% to 33.9%) and 3-year OS rate of 20.4% (95% CI = 17.6% to 23.2%), PD-1 blockade demonstrated higher 2-year OS (*P* < .001) and 3-year OS (*P* < .001) rates in the PD-L1 50% or greater subgroup, similar 2-year OS (*P* = .23) and 3-year OS (*P* = .38) rates in the PD-L1 1%-49% subgroup, and a lower 2-year OS rate (*P* = .003) in the PD-L1 less than 1% subgroup.

In second- or later-line therapy, treatment with PD-1 blockade resulted in 2-year OS and 3-year OS rates of 40.5% (95% CI = 37.6% to 43.5%) and 34.4% (95% CI = 30.5% to 38.3%), respectively, if PD-L1 was 50% or greater; 28.3% (95% CI = 22.1% to 34.5%) and 18.5% (95% CI = 13.2% to 23.9%), respectively, if PD-L1 was 1%-49%; and 25.4% (95% CI = 22.0% to 28.8%) and 13.7% (95% CI = 10.2% to 17.1%), respectively, if PD-L1 was less than 1% (Figure 4, B and D). Compared with docetaxel chemotherapy, which resulted in 2-year OS and 3-year OS rates of 16.7% (95% CI = 15.0% to 18.4%) and 9.1% (95% CI = 7.3% to 11.0%), respectively, PD-1 blockade in previously treated patients demonstrated statistically significantly higher 2-year OS and 3-year OS if PD-L1 was 50% or greater, 1%-49%, or less than 1%.

### Heterogeneity Testing

Because the number of studies being pooled for each estimate ranged from 1 to 18, and given that the fixed-effects approach yields consistent estimates of the underlying effect, the fixed-effect was the method of choice in terms of both numerical stability and interpretability given that heterogeneity was absent in the majority (>75% as calculated by Cochran’s Q test) of the pooled estimates (data not shown).

### Sensitivity Analyses Using Dako 22C3, Dako 28–8, or Ventana SP263 Studies Only

There was no difference in results when excluding studies that used SP142 and 73–10 assays and only including studies that used the 22C3, 28–8, and SP263 assays, which have the most comparable staining (Supplementary Figures 2-4, available online).

## Discussion

This meta-analysis provides a comprehensive summary of response rate and survival at key time points for treatment with PD-1 blockade monotherapy or chemotherapy in advanced NSCLC patients with various PD-L1 expressions. An increase in publications with longer term follow-up data from trials of PD-1 blockade in advanced NSCLC has provided the foundation for this meta-analysis, which builds on the work of previous meta-analyses on this topic (63–67) by including a number of newly published phase I-III trials, updated survival data from older trials, and survival outcomes based on survival at key time points rather than median survival.

A number of key practice points are suggested by the findings of this meta-analysis. Although there is increasing evidence in the first-line setting that combination PD-1 blockade plus chemotherapy with or without the antiangiogenesis agent bevacizumab or with or without a CTLA-4 inhibitor ipilimumab is the optimal treatment for patients with PD-L1 less than 50% or those with PD-L1 50% or greater with a high burden of disease (35,68–77), for patients unsuitable for combination therapy or those who prefer to spare chemotherapy toxicity, the choice between PD-1 blockade monotherapy and chemotherapy remains important. This meta-analysis supports the choice of PD-1 blockade over platinum doublet chemotherapy in patients with PD-L1 50% or greater, yielding higher ORR (39.7% vs 29.0%), 2-year PFS (24.6% vs 5.6%), and 3-year OS (38.3% vs 20.4%) rates. Patients with PD-L1 1%-49% are less likely to experience tumor response with PD-1 blockade than chemotherapy (16.6% vs 29.0%), but any clinical benefit is more likely to be sustained, demonstrated by higher 1-year PFS (35.0% vs 21.8%) and 2-year PFS (16.6% vs 5.6%) rates. This is an important distinction between a patient in whom urgent tumor shrinkage for symptom improvement is the primary goal of treatment compared with a patient who is asymptomatic and the chance of sustained disease control with minimal toxicity is the primary goal of treatment. The similar 2-year OS and 3-year OS rates between PD-1 blockade and chemotherapy in this group of patients, as shown in Figure 4, may be due to crossover of treatment with PD-1 blockade in later lines of therapy. In patients with PD-L1 less than 1%, most patients would benefit from chemotherapy rather than monotherapy PD-1 blockade because response rate (29.0% vs 8.3%) and 2-year OS rate (31.4% vs 16.7%) with chemotherapy are higher, and 1-year PFS rate is not any worse (21.8% vs 19.9%).

In previously treated patients, the chemotherapy tested in trials was docetaxel, which is considerably less effective than platinum doublet chemotherapy used in the first-line setting and is therefore a weaker comparator with PD-1 blockade. This meta-analysis concludes that PD-1 blockade provides equivalent or higher ORR, higher 1- and 2-year PFS rates, and higher 2- and 3-year OS rates than chemotherapy regardless of PD-L1 status. Even in patients with PD-L1 less than 1%, clinicians can expect a similar ORR to chemotherapy of approximately 10% but superior 1- and 2-year PFS rates and 2- and 3-year OS rates with PD-1 blockade. Therefore, our meta-analysis supports current clinical practice guidelines that recommend the use of nivolumab and atezolizumab irrespective of PD-L1 and the use of pembrolizumab for patients with PD-L1 1% or greater, based on randomized controlled trials that demonstrate superiority of these agents over docetaxel in their respective PD-L1 populations (5,12–15,78,79).

The findings from this meta-analysis demonstrate the continuous relationship between PD-L1 expression and outcomes from PD-1 blockade. There does not seem to be a natural cutpoint above which PD-1 blockade is definitely effective and below which PD-1 blockade is ineffective. This meta-analysis therefore does not support the use of a single cutpoint to classify patients as either “PD-L1 high” or “PD-L1 low” because it can falsely separate patients close to but on either side of a cutpoint, and falsely group on the same side of a cutpoint patients who may have very different PD-L1 expression. For example, to treat a patient with PD-L1 of 40% very differently from a patient with PD-L1 of 60%, when in fact they likely would have similar responses to PD-1 blockade, can be a consequence of dichotomizing patients according to a 50% cutpoint. Throughout other branches of medicine, it has been shown that when a continuous variable is interpreted as a dichotomous variable, there can be loss of information, unproven assumption of linearity, and reduction in statistical power to determine a true relationship between the variable and its outcome (80). If more studies were to report outcomes by PD-L1 expression as a continuous variable, or at least by centiles rather than overlapping subgroups, our understanding of the true relationship between PD-L1 expression and outcomes from PD-1 blockade would be more precise. This may also create better opportunity to compare outcomes against other treatment options across the whole spectrum of the continuous PD-L1 scale without being restricted if data have only been reported by a few selected cutpoints.

This meta-analysis demonstrates the benefit of reporting survival at key time points, which can inform clinical decisions that are of direct benefit to patients. The chance of prolonged survival is an important motivator for clinicians and patients to choose immunotherapy (81), and therefore reporting the proportion of patients in the “tail of the survival curve” provides key information for clinical decision making. As more studies report survival at key time points, future meta-analyses will become more powerful.

One limitation of this meta-analysis is the inclusion of nonrandomized trials including single-arm studies to achieve an adequate sample size when comparing PD-1 blockade and chemotherapy across various PD-L1 cutpoints. Comparison of outcomes between different arms of nonrandomized trials can be affected by different baseline patient characteristics and inter-trial protocol variability. Another limitation is the lack of uniformity of follow-up across trials that introduce variation in data maturity at 2-year and 3-year time points. Furthermore, publication bias is a risk, because trials showing poorer results from PD-1 blockade may be less likely to be published and updated. This meta-analysis also does not make comparison with the combinations of PD-1 blockade with chemotherapy, PD-1 blockade with cytotoxic T-lymphocyte-associated protein 4 (CTLA-4) inhibitors, or PD-1 blockade with chemotherapy and vascular endothelial growth factor (VEGF) inhibitors, which are important treatment options in the current landscape.

This study does not take into consideration a variety of other biomarkers that may also affect the efficacy of PD-1 blockade. The complexity of the immune oncology landscape is becoming increasingly evident, including the potential value of tumor mutational burden (82), immune-related gene expression signature (83,84), tumor-infiltrating lymphocyte density (85), programmed cell death ligand-2 (86), and many others.

Although this meta-analysis focuses on treatment efficacy, PD-1 blockade and chemotherapy also have different toxicity profiles, which are crucial in clinical decision making between the 2 treatment modalities. Previous meta-analyses have summarized these toxicity differences and contribute to informed treatment choices (87–89). Another area of further research includes the effect of PD-L1 expression on toxicity from PD-1–blocking drugs.

Evidently, PD-L1 expression is not a perfect predictor of outcomes from PD-1 blockade in advanced NSCLC but remains an important, most studied, and validated biomarker that has clear practical implications for treatment decisions.

## Funding

This research did not receive any specific grant from funding agencies in the public, commercial, or not-for-profit sectors.

## Notes

**Role of the funder:** Not applicable.

**Disclosures:** Johnathan Man’s disclosures are: Speaker honorarium: Novartis. Rina Hui’s disclosures are: Advisory Board member for: AstraZeneca, Bristol-Myers Squibb, Eli Lilly, Merck Sharp & Dohme, Novartis, Roche. Speaker honorarium from: Merck Sharp & Dohme, Novartis, Roche. The remaining authors have no disclosures.

**Author contributions:** Johnathan Man: Conceptualization, Methodology, Formal analysis, Investigation, Data Curation, Writing—Original draft, Writing—Review and editing, Visualization. Jared Millican: Conceptualization, Verification, Data curation, Writing—Review and editing. Arthur Mulvey: Conceptualization, Verification, Data curation, Writing—Review and editing. Val Gebski: Conceptualization, Methodology, Formal analysis, Writing—Original draft, Writing—Review and editing. Rina Hui: Conceptualization, Methodology, Writing—Review and editing, Supervision.

## Data Availability

No new data were generated or analyzed in support of this research.

## Supplementary Material

pkab012_Supplementary_Data
