# Supplementary material for: Response Rate and Survival at Key Timepoints With PD-1 Blockade vs Chemotherapy in PD-L1 Subgroups: Meta-Analysis of Metastatic NSCLC Trials
Source: JNCI Cancer Spectr. 2021 Jan 27;5(3):pkab012. doi: 10.1093/jncics/pkab012 (PMC8160531; doi:10.1093/jncics/pkab012)

Supplementary Data

Supplementary Methods

Search Strategy

Database: MEDLINE(R) All including Epub Ahead of Print, In-Process & Other Non-Indexed Citations, Daily and Versions(R); Embase 1996 to present

Search performed on 2 December 2019

1. programmed death-ligand 1.mp.
2. pd-l1.mp.
3. 1 or 2
4. lung.mp.
5. 3 and 4
6. remove duplicates from 5
7. limit 6 to yr="2000 - 2019"

Supplementary Tables

Supplementary Table 1: Cochrane Risk of Bias for Included Randomised Trials

First author (Trial name)	Year	Random Sequence Generation (Selection bias)	Allocation Concealment (Selection bias)	Blinding of Participants and Personnel (Performance bias)	Blinding of Outcome Assesment (Detection bias)	Incomplete Outcome Data (Attrition bias)	Selective Reporting (Reporting bias)	Other bias
Carbone **(Checkmate 026)**[19]	2017	Low	Low	Low (Open label but unlikely to influence outcomes)	Low	Low	Low	Low
Brahmer **(Checkmate 017)**[12, 22-26]	2015	Low	Low	Low (Open label but unlikely to influence outcomes)	High (Investigator radiological assessment)	Low	Low	Low
Borghaei **(Checkmate 057)**[22-26]	2015	Low	Low	Low (Open label but unlikely to influence outcomes)	High (Investigator radiological assessment)	Low	Low	Low
Mok **(KEYNOTE 042)**[34, 35]	2019	Low	Low	Low (Open label but unlikely to influence outcomes)	Low	Low	Low	Low
Reck **(KEYNOTE 024)**[36, 37]	2016	Low	Low	Low (Open label but unlikely to influence outcomes)	Low	Low	Low	Low
Hersbt **(KEYNOTE 010)**[13, 42, 43]	2015	Low	Low	Low (Open label but unlikely to influence outcomes)	Low	Low	Low	Low
Theelen **(Pembro-RT control arm)**[44]	2019	Low	Low	Low (Open label but unlikely to	Low (Independent reviewer for radiology	Low	Low	Low

				influence outcomes)	RECIST, but independent reviewer not blinded)			
Levy **(CC-486-NSCL-001)**[46]	2019	Low	Low	Low	High (Investigator radiological assessment)	Low	Low	Low
Spigel **(IMpower110)**[47]	2019	Low	Low	Low (Open label but unlikely to influence outcomes)	High (Investigator radiological assessment)	Low	Low	Low
Rittmeyer **(OAK)**[15, 51]	2018	Low	Low	Low (Open label but unlikely to influence outcomes)	High (Investigator radiological assessment)	Low	Low	Low
Fehrenbacher **(POPLAR)**[14, 52]	2016	Low	Low	Low (Open label but unlikely to influence outcomes)	High (Investigator radiological assessment)	Low	Low	Low
Rizvi **(MYSTIC)**[53]	2018	Low	Low	Low (Open label but unlikely to influence outcomes)	Low	Low	Low	Low
Barlesi **(JAVELIN Lung 200)**[60]	2018	Low	Low	Low (Open label but unlikely to influence outcomes)	Low	Low	Low	Low

Supplementary Table 2: ROBINS-I Risk of Bias for Included Non-Randomised Trials

First author (Trial name)	Year	Bias due to confounding	Bias in selection of participants into the study	Bias in classification of interventions	Bias due to deviations from intended interventions	Bias due to missing data	Bias in measurement of outcomes	Bias in selection of the reported result	OVERALL RISK OF BIAS: Low / moderate / serious / critical[◊]
Gettinger **(Checkmate 012)**	2016	Low	Moderate (ECOG 0 or 1 only)	Low	Low	Low	Moderate (All outcomes were investigator assessed)	Low	Moderate
Rizvi **(Checkmate 063)**	2015	Low	Moderate (ECOG 0 or 1 only)	Low	Low	Low	Low	Low	Moderate
Hida **(ONO-4538-05)**	2017	Low	Moderate (ECOG 0 or 1 only)	Low	Low	Low	Low	Low	Moderate
Nishio **(ONO-4538-06)**	2017	Low	Moderate (ECOG 0 or 1 only)	Low	Low	Low	Low	Low	Moderate
Gettinger **(Checkmate 003)**	2015	Low	Moderate (ECOG 0 or 1 only)	Low	Low	Low	Moderate (All outcomes were investigator assessed)	Low	Moderate
Garon **(KEYNOTE 001)**	2015	Low	Moderate (ECOG 0 or 1 only)	Low	Low	Low	Low	Low	Moderate
Nishio **(KEYNOTE 025)**	2018	Low	Moderate (ECOG 0 or 1 only)	Low	Low	Low	Low	Low	Moderate
Peters **(BIRCH)**	2017	Low	Moderate (ECOG 0 or 1 only)	Low	Low	Low	Low	Low	Moderate
Spigel **(FIR)**	2018	Low	Moderate (ECOG 0 or 1 only)	Low	Low	Low	Moderate (All outcomes were investigator assessed)	Low	Moderate

Antonia **(NCT01693562)**	2019	Low	Moderate (ECOG 0 or 1 only)	Low	Low	Low	Low	Low	Moderate
Garassino **(ATLANTIC cohorts 2 and 3)**	2018	Low	Moderate (ECOG 0 or 1 only)	Low	Low	Low	Low	Low	Moderate
Papadimitrakopoulou **(Lung-MAP SWOG S1400A)**	2017	Low	Low	Low	Low	Unknown	Moderate (All outcomes were investigator assessed)	Low	Moderate
Gulley; Jerusalem; Verschraegen **(JAVELIN Solid Tumor)**	2017	Low	Moderate (ECOG 0 or 1 only)	Low	Low	Low	Low	Low	Moderate
Wu **(SHR-1210-201)**	2019	Low	Moderate (ECOG 0 or 1 only)	Low	Low	Low	No information	Low	Moderate

Supplementary Figure 1: 2yr OS rate and 3yr OS rate of Chemotherapy Arms in Randomised Trials when stratified by different PD-L1 subgroups

(A) Treatment naïve patients; (B) Previously treated patients

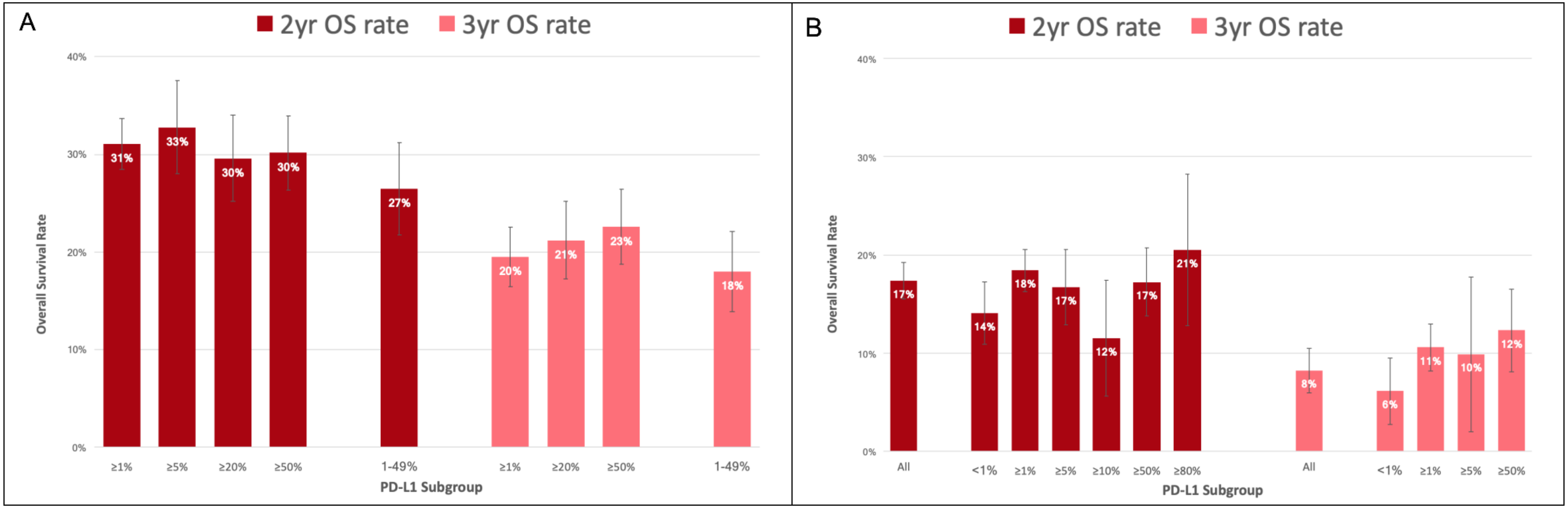

Supplementary Figure 2 (22C3, 28-8 and SP263 assays only)

(A) ORR in treatment naïve patients; (B) ORR in previously treated patients

⋆ = no available data

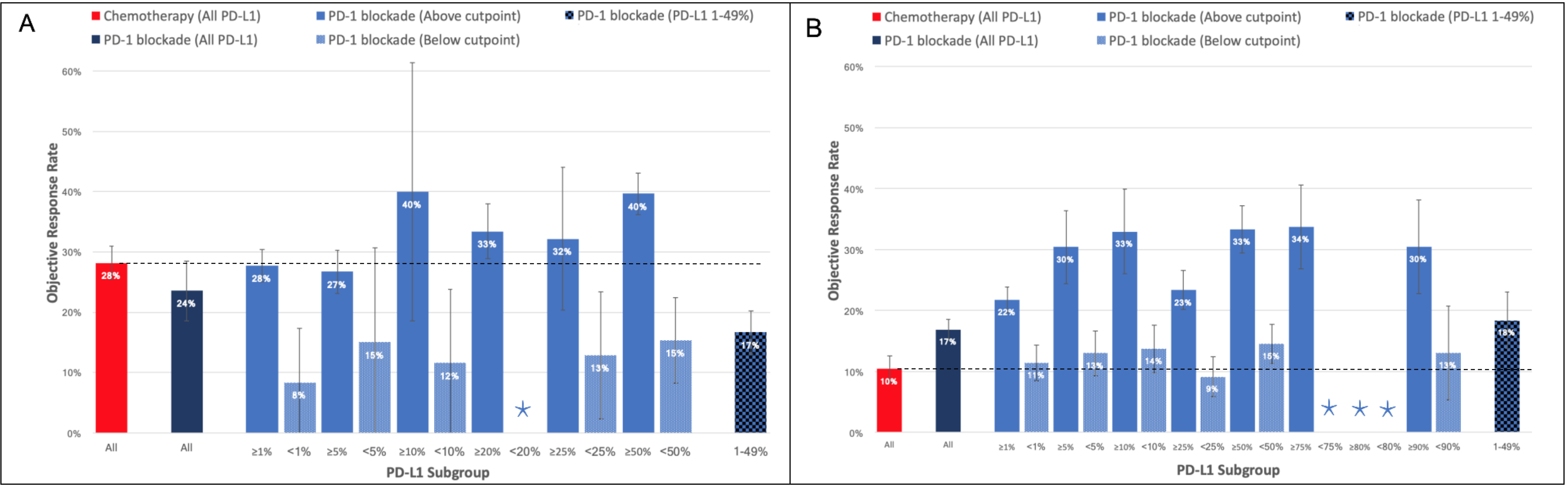

Supplementary Figure 3 (22C3, 28-8 and SP263 assays only)

(A) 1yr PFS in treatment naïve patients; (B) 1yr PFS in previously treated patients; (C) 2yr PFS in treatment naïve patients; (D) 2yr PFS in previously treated patients

✶ = no available data

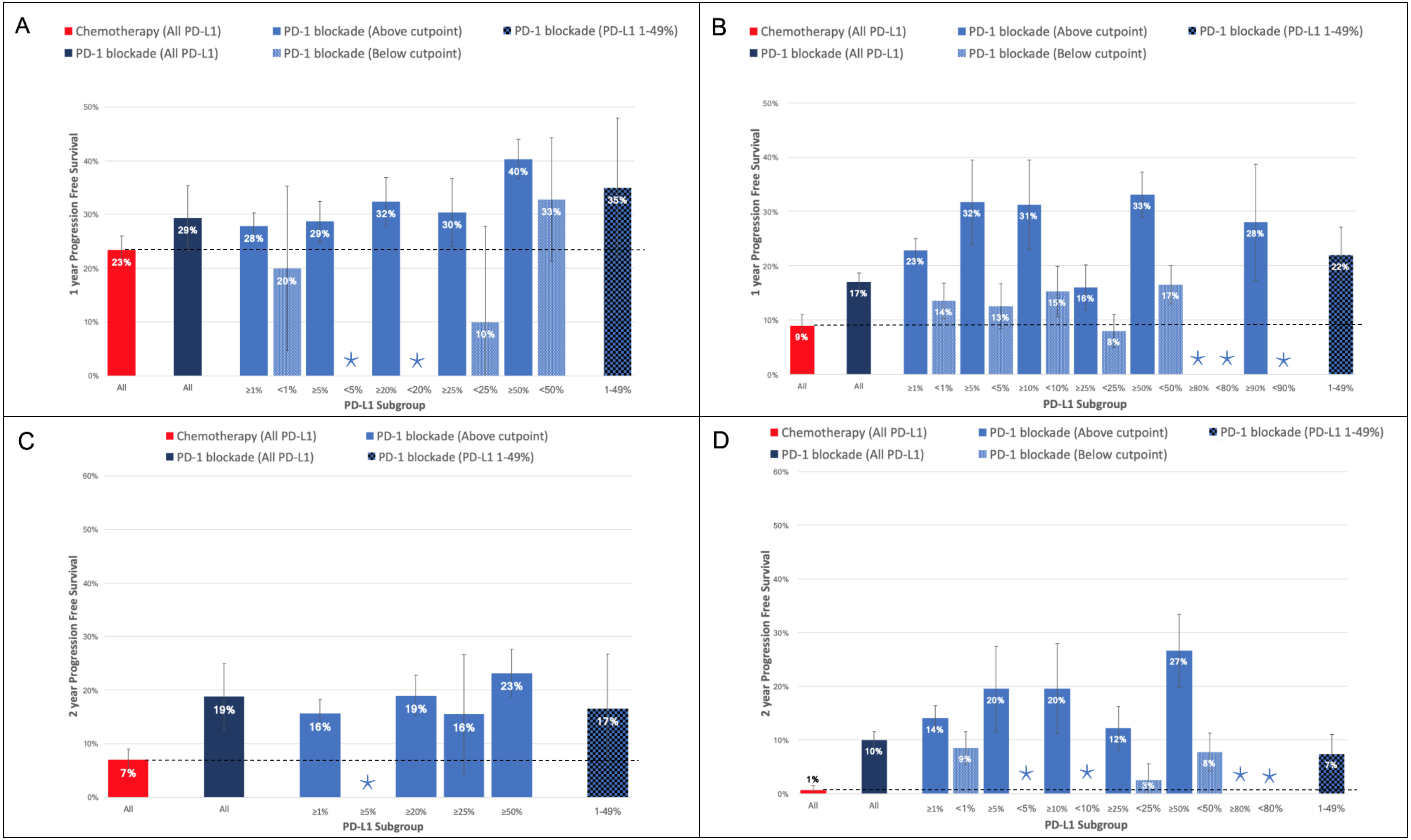

Supplementary Figure 4 (22C3, 28-8 and SP263 assays only)

(A) 2yr OS in treatment naïve patients; (B) 2yr OS in previously treated patients; (C) 3yr OS in treatment naïve patients; (D) 3yr OS in previously treated patients

⭑= no available data; # published 2yr OS and 3yr OS data for chemotherapy in treatment naïve patients did not include any patients with PD-L1 <1%

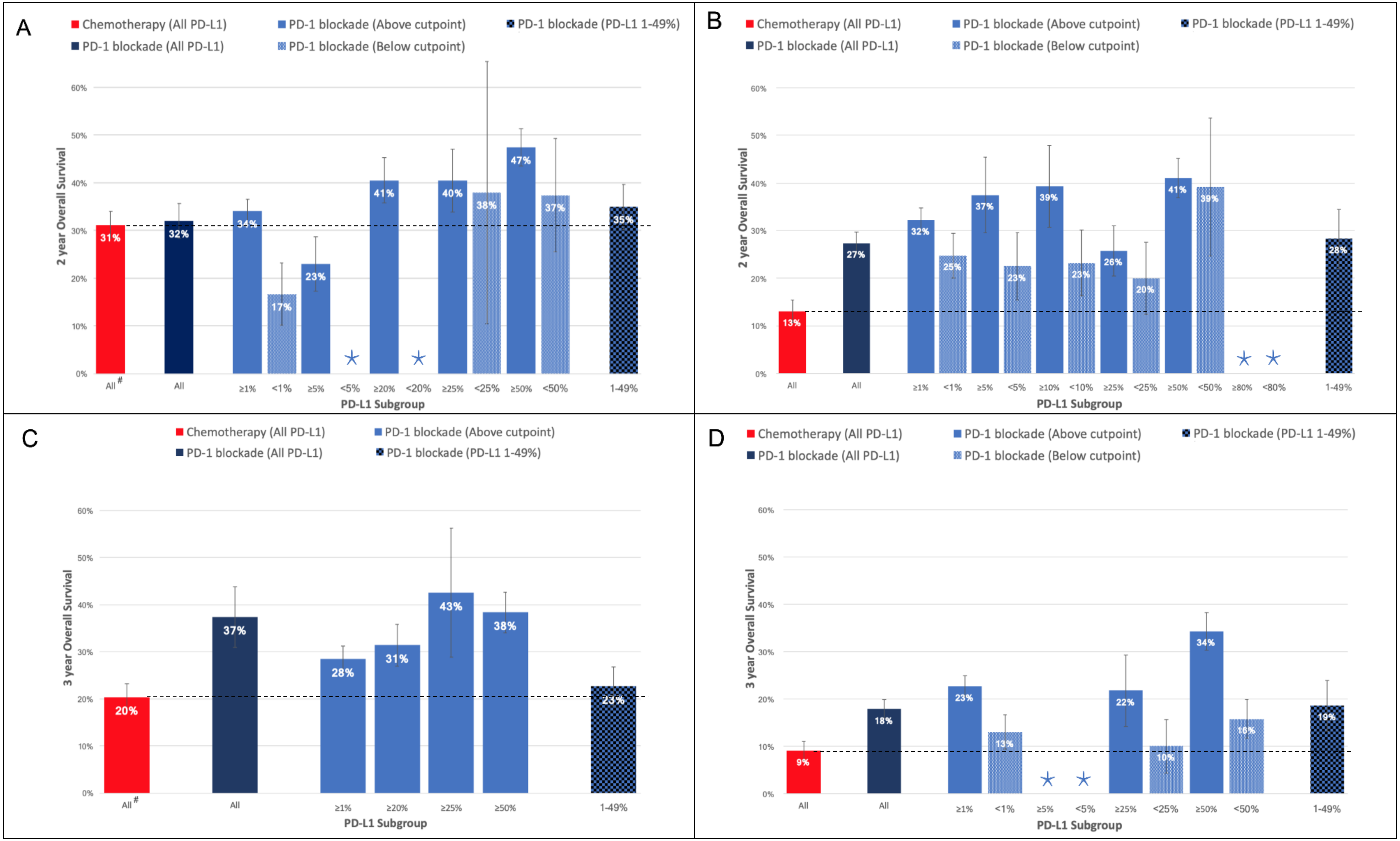

Supplement: pkab012_Supplementary_Data [file pkab012_supplementary_data.zip › CS20-0071R2 Man Supp mat_113020.pdf]
